# Immune responses and protection after DNA vaccination against *Toxoplasma gondii* calcium-dependent protein kinase 2 (*TgCDPK2*)

**DOI:** 10.1051/parasite/2017045

**Published:** 2017-11-09

**Authors:** Kai Chen, Jin-Lei Wang, Si-Yang Huang, Wen-Bin Yang, Wei-Ning Zhu, Xing-Quan Zhu

**Affiliations:** 1 State Key Laboratory of Veterinary Etiological Biology, Key Laboratory of Veterinary Parasitology of Gansu Province, Lanzhou Veterinary Research Institute, Chinese Academy of Agricultural Sciences, Lanzhou, Gansu Province 730046 PR China; 2 Jiangsu Co-innovation Center for Prevention and Control of Important Animal Infectious Diseases and Zoonoses, College of Veterinary Medicine, Yangzhou University, Yangzhou, Jiangsu Province 225009 PR China; 3 College of Veterinary Medicine, Northwest A&F University, Yangling, Shaanxi Province 712100 PR China; 4 College of Veterinary Medicine, Northeast Agricultural University, 59 Mucai Street, Harbin, Heilongjiang Province 150030 China

**Keywords:** *Toxoplasma gondii*, *TgCDPK2*, DNA vaccine, Protective immunity, Toxoplasmosis

## Abstract

*Toxoplasma gondii*, an intracellular zoonotic protozoan parasite, is possibly the most widespread parasite of warm-blooded animals and can cause serious public health problems and economic losses worldwide. *TgCDPK2*, a member of the *T. gondii* calcium-dependent protein kinase family, was recently identified as an essential regulator for viable cyst development in *T. gondii*. In the present study, we evaluated the protective immunity induced by DNA vaccination based on a recombinant eukaryotic plasmid, pVAX-TgCDPK2, against acute toxoplasmosis in mice. BALB/c mice were intramuscularly immunized with pVAX-TgCDPK2 plasmid and then challenged by infection with the highly virulent RH strain of *T. gondii*. The specific immune responses and protective efficacy against *T. gondii* were analyzed by cytokine and serum antibody measurements, lymphocyte proliferation assays, flow cytometric on lymphocytes and the survival time of mice after challenge. Our results showed that mice immunized with pVAX-TgCDPK2 could elicit special humoral and cellular responses, with higher levels of IgG antibody, and increased levels of Th1-type cytokines IFN-γ, IL-12(p70), and CD3 + CD4 + CD8 − and CD3 + CD8 + CD4 − T cells, and had a prolonged survival time (14.0 ± 2.32 days) compared to control mice. These results demonstrate that pVAX-TgCDPK2 is a potential vaccine candidate against acute toxoplasmosis.

## Abstract (fr)

*Toxoplasma gondii*, parasite protozoaire zoonotique intracellulaire, est probablement le parasite des animaux à sang chaud le plus répandu et peut représenter un problème majeur de santé publique et des pertes économiques dans le monde entier. *TgCDPK2*, l'une des protéines kinases dépendantes du calcium de *T. gondii*, a été récemment identifiée comme étant un régulateur essentiel du développement viable des kystes chez *T. gondii*. Dans la présente étude, nous avons évalué l'immunité protectrice induite contre la toxoplasmose aiguë chez la souris par la vaccination par ADN basée sur un plasmide eucaryote recombinant, pVAX-TgCDPK2. Des souris BALB/c ont été immunisées par voie intramusculaire avec le plasmide pVAX-TgCDPK2, suivi par une infection avec la souche RH de *T. gondii*, hautement virulente. Les réponses immunitaires spécifiques et l'efficacité protectrice contre *T. gondii* ont été analysées par des mesures de cytokines et d'anticorps sériques, des tests de prolifération lymphocytaire, la cytométrie de flux des lymphocytes et le temps de survie des souris après infection. Nos résultats ont montré que les souris immunisées avec pVAX-TgCDPK2 pouvaient susciter des réponses humorales et cellulaires particulières, avec un taux plus élevé d'anticorps IgG et des niveaux accrus de cytokines de type Th1, IFN-y, IL-12(p70) et CD3 + CD4 + CD8 - et CD3 + CD8 + CD4 -, et avaient un temps de survie prolongé (14,0 ± 2,32 jours) par rapport aux souris témoins. Ces résultats ont démontré que pVAX-TgCDPK2 était un candidat vaccin potentiel contre la toxoplasmose aiguë.

## Introduction

*Toxoplasma gondii*, an obligate intracellular apicomplexan parasite, can infect almost all warm-blooded animals, including humans [4,40]. It is estimated that one-third of the human population worldwide is chronically infected by this parasite [28]. During pregnancy, the infection may lead to serious symptoms such as abortion, stillbirth and significant congenital defects in the fetus [28]. In immune-compromized individuals, such as AIDS patients, transplant recipients and certain cancer patients, toxoplasmosis can cause severe disease sometimes with fatal outcomes [8,38]. The high prevalence of *T. gondii* in livestock can cause sizable economic losses in animal industries [35]. Moreover, infected animals may be a source of secondary infection since humans can be infected by consuming uncooked or undercooked meat from infected livestock [20]. Unfortunately, current chemotherapy against toxoplasmosis only targets the tachyzoite stage, while no effective drugs are recommended to treat the bradyzoite stage [5]. Therefore, vaccination was naturally considered as an alternative strategy to alleviate the burden of infection.

In recent years, DNA-based vaccines have been developed against *T. gondii* infection. With the ability of eliciting high levels of both humoral and cellular immune response, a DNA vaccine has been seen as the most promising [14,17]. Many DNA vaccine candidates have been evaluated and some of them have shown promise [7,21,24,31]. Thus, the identification of novel *T. gondii* antigens is important for research into DNA vaccination.

Currently, the calcium-dependent protein kinase (CDPK) family is the focus of interest because these enzymes are prominent in the calcium signaling cascades and have been widely identified in plants, ciliates and apicomplexans but are absent from fungi, animals and humans [13]. *T. gondii* contains 14 CDPKs which play important roles in the *Toxoplasma* life cycle including host cell invasion, egress, gliding motility, and replication [25,26,30,34]. Among the 14 members of the *T. gondii* CDPK family, CDPK1, CDPK3, CDPK5 and CDPK6 have been shown to elicit favorable immunogenicity against *T. gondii* [6,41–43], and are therefore considered promising candidate vaccines. *TgCDPK2* plays a critical role in amylopectin metabolism [34]. It was demonstrated that the loss of *TgCDPK2* results in the hyperaccumulation of amylopectin polymer and leads to gross morphological defects and inability of cyst formation in a mouse model [34]. However, the immunogenicity of TgCDPK2 and its suitability as a promising candidate vaccine have not been evaluated.

The objectives of this study were to evaluate the immunogenicity of *T. gondii* CDPK2 in BALB/c mice by constructing eukaryotic pVAX-TgCDPK2 plasmids, and to determine the protective ability of the DNA vaccine based on pVAX-TgCDPK2 against the highly virulent *T. gondii* RH strain in a BALB/c mouse model.

## Materials and methods

### Ethics statement

All animals were handled in strict accordance with good animal practice according to the Animal Ethics Procedures and Guidelines of the People's Republic of China, and the study was approved by the Animal Administration and Ethics Committee of Lanzhou Veterinary Research Institute, Chinese Academy of Agricultural Sciences (Permit No. LVRIAEC-2009-006).

### Experimental mice and parasites

Specific pathogen-free (SPF) female BALB/c mice of six-to-eight-weeks of age were purchased from the Center of Laboratory Animals, Lanzhou Institute of Biological Products, Lanzhou, China. Tachyzoites of the *T. gondii* RH strain (type I) were maintained in our laboratory and prepared from human foreskin fibroblast (HFF) cells, and HFF cells were cultured with Dulbecco's modified Eagle's medium (DMEM) supplemented with 2% fetal bovine serum (FBS) (Gibco, Carlsbad, CA, USA).

### Cloning and molecular characterization of *TgCDPK2*

The total RNA of tachyzoites of the RH strain was extracted using Trizol reagent (Invitrogen, USA) according to the manufacturer's instructions. A fragment of the *TgCDPK2* gene (ToxoDB accession ID: TGME49_225490) of *T. gondii* was amplified by RT-PCR, using the following primers: forward: 5'-GGTACCATGCCGCTCAAGACTTCCTGG3' and reverse: 3'-TCTAGATTACC- CCGTAGCGCGAGGCG5' containing the *Kpn* I and *Xba* I restriction sites. The amplicon was then inserted into the pMD18-T vector (TaKaRa, Japan). The *TgCDPK2* fragment cleaved from pMD-TgCDPK2 was sub-cloned into the pVAX I vector (Invitrogen, USA) using T4 DNA ligase to construct a recombinant plasmid named pVAX-TgCDPK2. The recombinant plasmids were identified by PCR, double restriction enzyme digestion and sequencing. The positive plasmids were purified from transformed *Escherichia coli* DH5α cells by anion exchange chromatography and tested by spectrophotometer to determine the concentrations, and dissolved in sterile phosphate-buffered saline (PBS) with a final concentration of 1 µg/µL and stored at − 20°C until use.

### Expression and analysis of pVAX-TgCDPK2 *in vitro*

The recombinant pVAX-TgCDPK2 plasmids were transfected into HEK 293-T cells using the LipofectamineTM 2000 reagent (Invitrogen, USA), as indicated by the manufacturer. Forty-eight hours after transfection, the expression of pVAX-TgCDPK2 was examined by indirect immunofluorescence (IFA). Briefly, HEK 293-T cells were fixed with 4% paraformaldehyde and then incubated with goat anti-*T. gondii* tachyzoites polyclonal antibody (1:50). Then, fluorescein isothiocyanate (FITC)-labeled rabbit anti-goat IgG antibody (1:1000; Sigma) was added. The specific fluorescence was imaged through a Zeiss Axioplan fluorescence microscope (Carl Zeiss, Germany). As a negative control, the HEK 293-T cells were transfected with pVAX I.

### DNA immunization and challenge infection

Eighty female BALB/c mice were randomly divided into four groups (20 mice in each group). For the experimental group, mice were immunized by bilateral intramuscular injection into the quadriceps three times at two-week intervals with 100 µL (1 µg/µL) pVAX-TgCDPK2. As negative controls, two groups were injected with 100 µL (1 µg/µL) empty pVAX I vector or 100 µL PBS, respectively, and the fourth group received nothing as a blank control. Serum samples were collected from the tail vein from 3 mice in each group on the day before each immunization, and stored at −20°C for further analysis.

Two weeks after the last immunization, 10 mice in each group were challenged intraperitoneally with 1×10^3^ tachyzoites of highly virulent *T. gondii* RH strain. The number of surviving mice was recorded each day.

### Evaluation of humoral responses

The levels of specific antibodies (IgG, IgG1 and IgG2a) in mice sera were determined by ELISA, as described previously [36]. 96-well microtiter plates were first coated with 100 µL STAg (10 µg/mL) at 4°C overnight and then washed three times with 0.05% Tween 20 in PBS (PBST) and blocked with PBS containing 1% BSA at room temperature for 1 h. The plates were washed three times with PBST and then incubated with mouse serum sample (1:50 dilutions) at room temperature for 1 h. After washing five times with PBST, the wells were incubated with horseradish peroxidase (HRP) conjugated anti-mouse IgG (1:250 dilutions), IgG1 or IgG2a (1:500 dilutions) at room temperature for 1 h, respectively. After washing six times with PBST and incubation with 100 µL substrate solution (pH = 4.0) (1.05% citrate substrate buffer; 1.5% ABTS; 0.03% H_2_O_2_) for 20 min, the reaction was stopped by adding 50 µL 1M H_2_SO_4_, the absorbance was measured at 450 nm (Bio-TekEL x 800, USA). All tests were carried out in triplicate.

### Lymphocyte proliferation assays

Two weeks after the last immunization, lymphocyte proliferation assays were performed using the Enhanced Cell Counting Kit-8 (Beyotime, China). Three mice from each group were sacrificed to harvest the spleens. Splenocytes were obtained aseptically via mechanical filtration of the splenic organ with a 200 mesh sieve, and red blood cells were lyzed by erythrocyte lysis buffer (0.15 M NH_4_Cl, 1.0 M KHCO3, 0.1 mM EDTA, pH 7.2). The splenocytes were resuspended in Dulbecco's modified Eagle's medium (DMEM) supplemented with 2% fetal bovine serum (FBS) and were then cultured in 96-well microtiter plates in triplicate, at a density of 2 × 10^5^ cells per well. Lymphocytes were stimulated with STAg (10 μg/mL) or concanavalin A (ConA; 5 μg/mL; Sigma), or medium alone served as positive and negative controls, respectively. After incubation at 37°C in a 5% CO_2_ for 44 h, to each well we added 10 µL CCK-8 reagent (provided by Enhanced Cell Counting Kit-8, Beyotime, China), and incubation continued for a further 4 h. The proliferative activity was evaluated by measuring absorbance at 570 nm. The stimulation index (SI) for each group was calculated as follows: (OD_570_STAg/OD_570_Control) : (OD_570_ConA/OD_570_Control).

### Cytokine assays

Splenocytes harvested from each group were cultured with STAg or medium alone (negative control) in flat-bottom 96-well microtiter plates, as described in the section on the lymphocyte proliferation assay. Cell-free supernatants were collected and assayed for the level of IL-4 at 24 h, IL-10 at 72 h, and IFN-γ and IL-12(p70) at 96 h using commercial ELISA kits according to the manufacturer's instructions (Biolegend, USA) and previous studies [41,43]. The analysis was performed in three independent experiments.

### Flow cytometry analysis

Splenic lymphocytes were harvested as described above, viability was determined using 0.04% trypan blue (viability > 90%) and cell concentration was adjusted to 1 × 10^6^ cells/mL in PBS containing 2% FBS. As in a previous study [37], the lymphocytes were incubated with surface markers including phycoerythrin (PE)-labeled anti-mouse CD3, allophycocyanin (APC)-labeled anti-mouse CD4 and fluorescein isothiocyanate (FITC)-labeled anti-mouse CD8 (eBioscience) at 4°C for 30 min in the dark. Then, the cells were fixed with FACScan buffer (PBS containing 1% FCS, 0.1% sodium azide and 2% paraformaldehyde). The analysis of surface markers (CD3, CD4 and CD8) on the cells was performed with fluorescence profiles through a FACScan flow cytometer (BD Biosciences, USA).

### Statistical analysis

All statistical analyses were performed by SPSS18.0 Data Editor (SPSS Inc., Chicago, IL, USA). The differences in antibody responses, lymphoproliferation assays, cytokine production, and percentages of CD3 + CD4 + CD8− and CD3 + CD8 + CD4 − T cells, between all the groups were analyzed by one-way ANOVA, and the difference were considered statistically significant if *p* < 0.05.

## Results

### Expression of pVAX-TgCDPK2 plasmid *in vitro*

**Figure 1:**
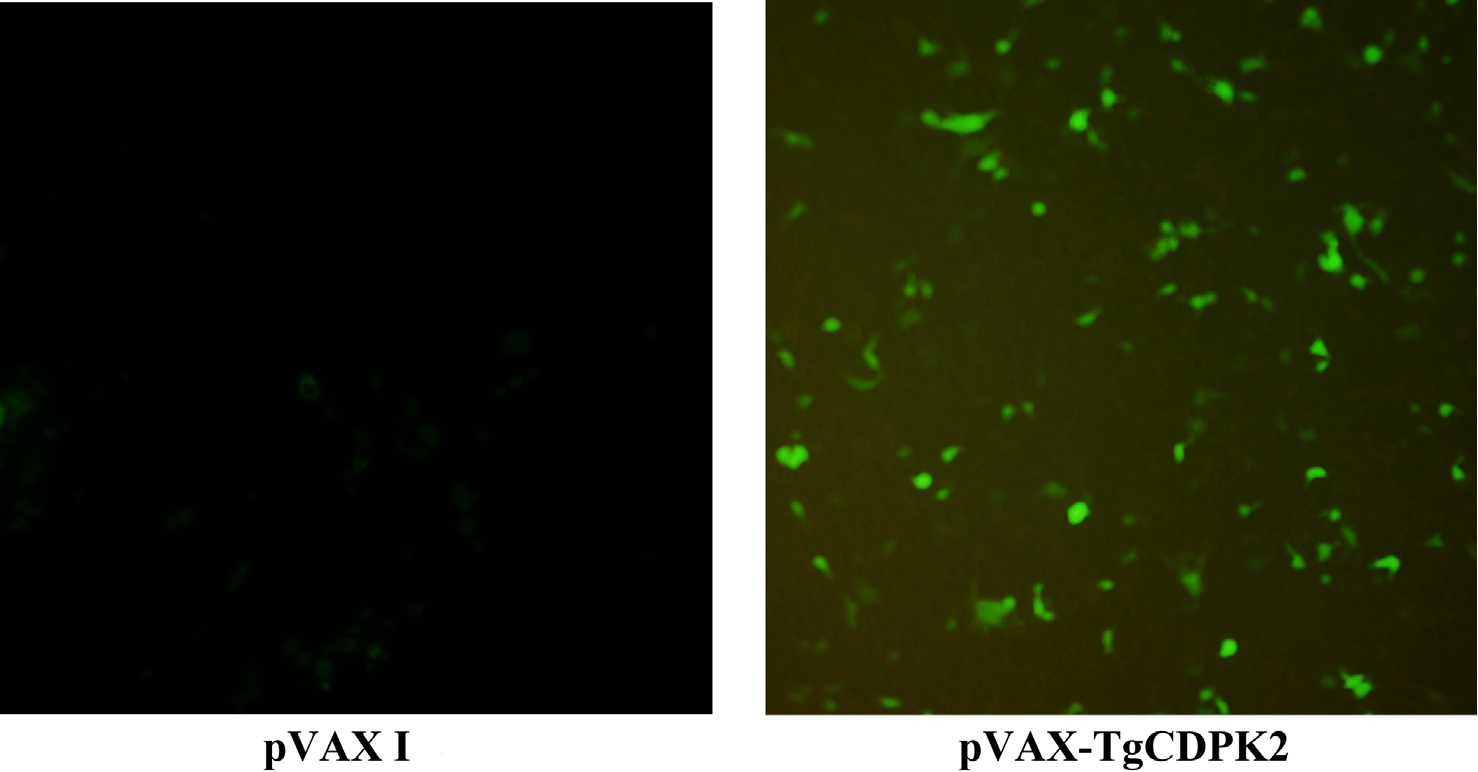
Indirect immunofluorescence detection of *TgCDPK2* expression on HEK 293-T cells at 48 h post-transfection. A. HEK 293-T cells transfected with empty vector pVAX I; B. HEK 293-T cells transfected with pVAX-TgCDPK2.

The expression of *TgCDPK2* in HEK 293-T was detected by indirect immunofluorescence (IFA). The results showed that specific green fluorescence was detected in HEK 293-T cells transfected with pVAX-TgCDPK2, while no green fluorescence was observed in the negative control cells (transfected with the pVAX I plasmids) (Fig. 1). These data indicate that the TgCDPK2 protein was successfully expressed in the HEK 293-T cells.

### Specific IgG and IgG isotypes induced by TgCDPK2

**Figure 2:**
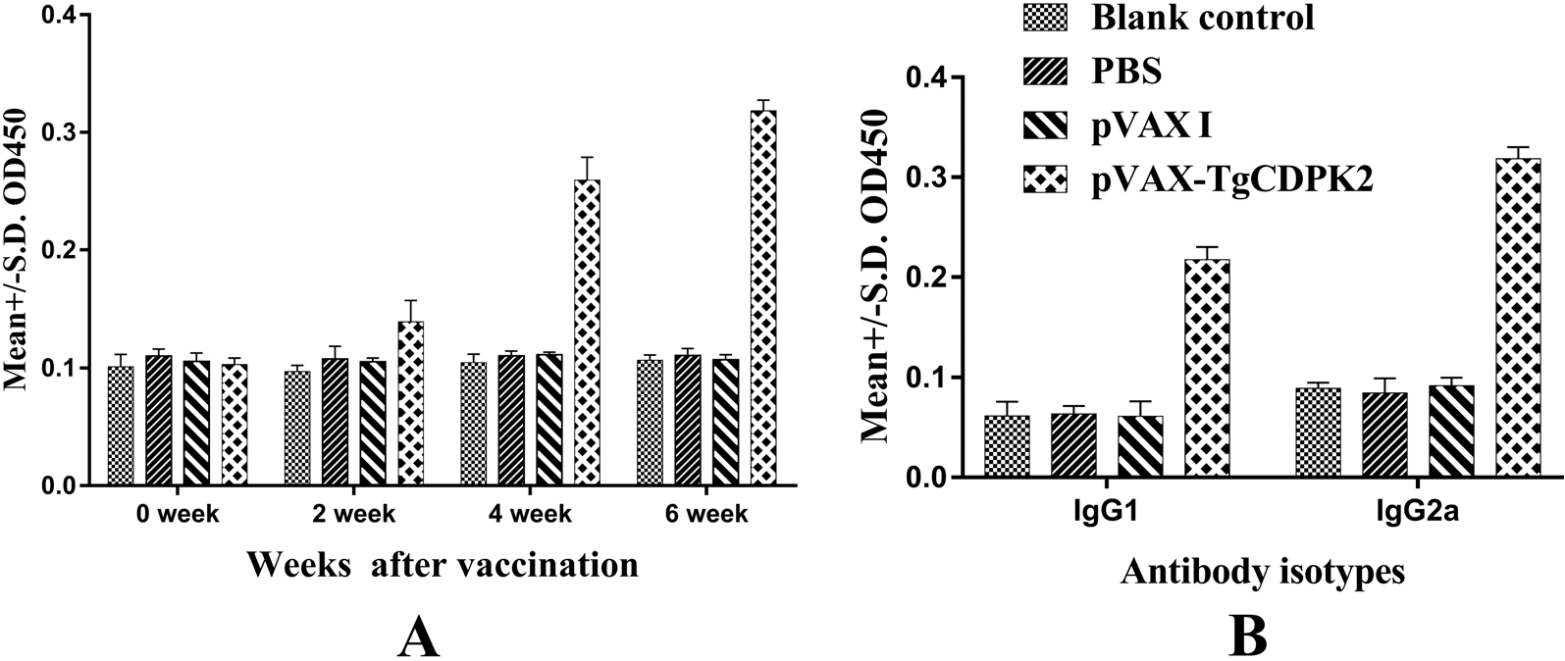
Humoral response in BALB/c mice induced by DNA vaccination A. Determination of specific IgG antibodies in the sera of BALB/c mice at 0, 2, 4 and 6 weeks. B. Determination of the specific IgG antibody subclass profile (IgG1 or IgG2a) in the sera of BALB/c mice 2 weeks after the third immunization. Results are expressed as means of the OD405 ± SD (n = 3), and statistically significant differences (*p* < 0.05) are indicated by (*).

In order to evaluate the levels of specific antibodies against *T. gondii*, the serum samples from immunized and control groups were detected. The results indicate that immunization induced a significant IgG response in the immunized group compared to the PBS, pVAX I or blank control groups (*p* < 0.05) (Fig. 2A). For mice in the three control groups (PBS, pVAX I or blank control groups), specific antibody levels did not significantly increase with the continuous immunization (*p*>0.05).

To characterize the Th1 and/or Th2 response in the immunized mice, the expression level of subclasses of IgG (IgG1 and IgG2a) was analyzed individually in sera of mice from all groups at two weeks after the last immunization. As shown in Fig. 2B, the levels of IgG1 and IgG2a in the pVAX-TgCDPK2 immunized group were significantly higher compared with the three control groups (blank control, PBS and pVAX I controls) (*p* < 0.05). These results suggest that the specific humoral response with a mixed Th1/Th2 profile was elicited by the DNA immunization, with a predominant Th1 type immune response in mice immunized with pVAX-TgCDPK2.

### Lymphocyte proliferation assay

The assay was carried out to analyze the proliferation of splenocytes stimulated by STAg or ConA at two weeks after last immunization. The assay results rendered in the proliferation stimulation index (SI) are summarized in Table 1. The SI was 1.76 ± 0.24 in the pVAX-TgCDPK2 immunized group, which was significantly higher than that in the three control groups (blank control, PBS and pVAX I controls) (*p* < 0.05), while the three control groups presented no significant differences (*p* > 0.05).

**Table 1:** Splenocyte proliferative responses and the percentages of CD3+CD4+CD8- and CD3+CD8+CD4- T cells in immunized mice 2 weeks after the last pVAX-CDPK2 immunization.

Group	SI (Mean ± SD)	CD3 + CD4 + CD8 -(%)	CD3 + CD8 + CD4 -(%)
pVAX-TgCDPK2	1.76 ± 0.24^*^	20.71 ± 0.68^*^	6.20 ± 0.58^*^
pVAX I	1.03 ± 0.15	10.51 ± 3.00	3.30 ± 0.60
PBS	1.05 ± 0.16	9.12 ± 2.39	3.21 ± 0.95
Blank control	1.16 ± 0.12	12.07 ± 2.56	3.66 ± 0.13

Note: statistically significant differences (*p* < 0.05) between groups are indicated by ^*^

### Flow cytometry analysis

The percentages of CD3 + CD4 + CD8− and CD3 + CD8 + CD4− T cells in the spleen of mice from each group were analyzed by flow cytometry and results are summarized in Table 1. In the mice immunized with pVAX-TgCDPK2, the percentage of CD3 + CD4+CD8 − was 20.71% and CD3+CD8+CD4 − was 6.20%, values which were significantly increased compared to the control groups (*p* < 0.05). However, the percentages of these two T-cell subtypes were not significantly different among the control groups (*p*>0.05).

### Cytokine production

Supernatants were collected from splenocytes of individual mice at 2 weeks after the last immunization, cultured under STAg stimulation, and were used to examine the levels of IL-4, IL-10, IFN-γ and IL-12(p70) by ELISA. As shown in Table 2, compared to the three control groups, the levels of IFN-γ, IL-12(p70) and IL-10 in spleen cell cultures from the mice immunized with pVAX-TgCDPK2 increased significantly (*p* < 0.05). However, the level of IL-4 in splenocyte cultures from the pVAX-TgCDPK2 immunized group showed no significant difference with that in the three control groups (*p*>0.05).

**Table 2:** Cytokine productions of splenocytes induced by soluble tachyzoite antigens of *T. gondii*

Group	Cytokine production (pg/mL)			
	
	IFN-γ	IL-12(p70)	IL-10	IL-4
pVAX-TgCDPK2	694.56 ± 76.72^*^	334.51 ± 7.52^*^	421 ± 23.25^*^	11.75 ± 2.98
pVAX I	104.63 ± 28.39	< 15	< 15	11.29 ± 4.45
PBS	89.14 ± 20.14	< 15	< 15	13.17 ± 0.98
Blank control	62.14 ± 26.92	< 15	< 15	11.67 ± 3.21

Note: statistically significant differences (*p* < 0.05) between groups are indicated by ^*^

### Protective efficacy in immunized mice

**Figure 3:**
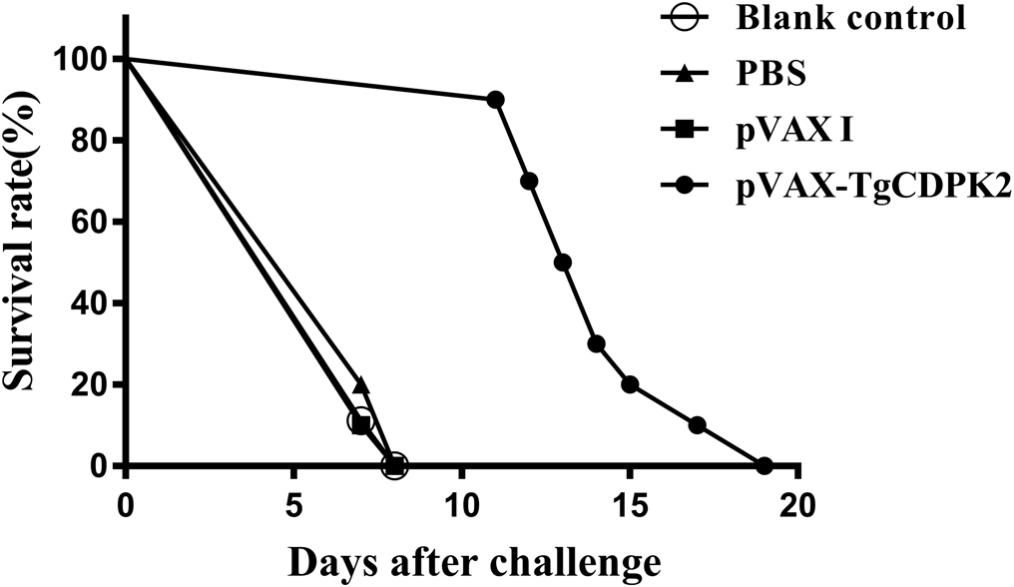
Protection of BALB/c mice against *T. gondii* infection. Survival time of mice immunized with PBS, pVAX I, pVAX-TgCDPK2 or blank control after challenge with 1×10^3^ tachyzoites of *T. gondii* RH strain. Each group had 10 mice. All the mice in control groups (PBS, pVAX I, blank control) died at day 7.

In order to evaluate the protective immunity induced by immunization with pVAX-TgCDPK2 plasmids, ten mice from each group were challenged intraperitoneally with 1×10^3^ tachyzoites of the virulent *T. gondii* RH strain at 2 weeks after the last immunization. The survival curves for all groups of mice are shown in Fig. 3. The difference in the average survival time of mice among the three control groups was not statistically significant: all mice died within 8 days after challenge (blank control, 7.11 ± 0.33 days; PBS control, 7.22 ± 0.44 days; pVAX I control, 7.11 ± 0.33 days) (*p>*0.05). The average survival time of mice immunized with pVAX-TgCDPK2 (14 ± 2.32 days) was significantly longer than that of the three control groups (*p *< 0.05). However, immunization with pVAX-TgCDPK2 could not completely protect the immunized mice from death.

## Discussion

Over the last few years, DNA vaccines have shown potential to induce resistance against *T. gondii* infection by eliciting long-term humoral and cellular immune responses in animal models, which is critical for protective immunity against *T. gondii* infection [3,22,32,36,39,46,47].

CDPKs are vital mediators of Ca^2+^ signaling and participate in parasite host cell egress, invasion, extracellular motility and cell division [25,26,30,34]. Since CDPKs are absent in mammals, the CDPK family has been considered a promising target for anti-*T. gondii* drugs and an ideal candidate antigen for vaccines [6,41–43]. Recently, Uboldi et al reported that CDPK2 is associated with the formation of bradyzoites in *T. gondii* in that the deletion of CDPK2 gene can lead to massive ultrastructural changes and loss of viability in the bradyzoite stage [34], suggesting that CDPK2 could be selected as an alternative antigen for a vaccine against *T. gondii*. In this study, we evaluated the immunogenicity and protective efficacy of a DNA vaccine based on the *TgCDPK2* gene against acute toxoplasmosis in a BALB/c mouse model. Our results indicate that vaccination of BALB/c mice with pVAX-TgCDPK2 can induce high levels of specific humoral and cellular immune responses, resulting in effective protective immunity, showing increased survival time (14 ± 2.32 days, *p* < 0.05), which demonstrates that *TgCDPK2* is a promising DNA vaccine candidate against acute toxoplasmosis.

When *T. gondii* invasion occurs for the first time *in vivo*, the parasite can be captured and processed by antigen-presenting cells and then presented to T lymphocytes, which further build adaptive immunity. When *T. gondii* invade subsequently, special anti-*T. gondii* IgG antibodies adhere to the surface of parasites and limit their spread by preventing attachment to host cell receptors, resulting in their elimination by macrophages [9]. Therefore, humoral response, by promoting macrophages to kill intracellular parasites, has been considered to be of great importance in immunity against *T. gondii* infection. In the present study, we evaluated humoral response intensity on the basis of specific anti-*T. gondii* IgG levels. The detected IgG levels in mice successively immunized with pVAX-TgCDPK2 were significantly higher than those in the control groups, suggesting that successful and continuous humoral immunity was induced by TgCDPK2.

High levels of IgG1 and IgG2a were detected in the serum of mice in the immunized group compared to the control groups. This result was consistent with previous studies, showing that DNA immunization could elicit a mixed Th1/Th2 immune response, with a more significant IgG2a response [1,44,45]. The higher ratio of IgG2a/IgG1 in mice immunized with pVAX-TgCDPK2 shows that a predominant Th1-type immune response was elicited. A series of studies have revealed that IFN-γ primarily produced by NK- or T-cells can restrict the growth of *T. gondii* in the acute or chronic phase of infection and that IFN-γ signaling plays a critical role in activating anti-microbial inducible effectors important for the control of intracellular parasites through the transcription factor STAT1 [11,23]. Meanwhile, the production of IL-12 by accessory cells can promote the ability of NK- and T-cells to produce IFN-γ [16,27,33]. These findings confirmed the significance of the cooperation between IFN-γ and IL-12 in anti-*T. gondii* infection. In this study, the high levels of IFN-γ and IL-12 in the supernatant of splenocytes of mice immunized with pVAX-TgCDPK2 suggest that intense Th1-type responses were elicited. The enhanced level of IFN-γ and IL-12 further plays an important role in promoting lymphocyte differentiation and enhancing the ability of NK-cells to kill *T. gondii* [2,18,19]. In addition, IL-10 was also highly increased in immunized mice. As a regulatory cytokine, the increased level of IL-10 is able to regulate the Th1-type response which may lead to potential immunopathological mechanisms with high levels of IFN-γ production [12,15,29]. Therefore, the high IL-10 level contributed to the longer survival time of mice immunized with pVAX-TgCDPK2. IL-4 is critical in the early phase of acute *T. gondii* infection, which partially contributed to protection against the acute stage of *T. gondii* infection. However, in the present study, the expression of IL-4 in mice immunized with pVAX-TgCDPK2 was not significantly elicited compared to the three control groups, suggesting that immunization with pVAX-TgCDPK2 cannot promote related cell responses. This may explain why the DNA vaccine was not able to provide complete protection of mice against acute *T. gondii* infection.

Cellular immunity plays an important role in the control of *T. gondii* infection. In this study, a significantly higher level of splenocyte proliferative response was induced by DNA immunization with pVAX-TgCDPK2. This suggests that cellular immune response was elicited in the immunized mice. The flow cytometry analysis indicated that the percentages of both CD3 + CD4 + CD8 − and CD3 + CD8 + CD4 − were significantly higher in the mice in the experimental group compared to the three control groups, and the results were similar to those induced by DNA vaccination with *CDPK3, CDPK5*, and *ROP18* of *T. gondii* [41-43]. These increased CD3 + CD4 + CD8 − and CD3 + CD8 + CD4 − T cell levels will increase the cytotoxic activity against *T. gondii* infection. Furthermore, the proliferation stimulation index of splenocytes in the experimental group was significant higher in contrast to control groups. It revealed that a large amount of memory lymphocytes were produced after being stimulated by the immunization, which will help to protect against infection by *T. gondii*.

In the present study, we found that intramuscular immunization with pVAX-TgCDPK2 can induce partial immune protection, and the immunized mice had a prolonged survival time compared to the control groups when challenged with high doses of *T. gondii* RH strain. Considering that DNA vaccines are usually based on a single antigen and are largely limited by the major histocompatibility complex (MHC) [10], pVAX-TgCDPK2 may not readily induce an effective and complete immune response against *T. gondii* infection. In future studies, a higher quantity of *T. gondii* antigen combined with an efficient adjuvant could be explored to enhance the protective efficacy.

In conclusion, this study explored the feasibility of a novel vaccine candidate, *TgCDPK2*, for protection against *T. gondii* infection. The vaccine candidate was able to induce strong cellular and humoral immune response, and to provide partial protection against acute toxoplasmosis.

### Competing interests

The authors declare that they have no competing interests.
